# Analytic and Diagnostic Validation of a Targeted Next-Generation Sequencing Panel for Common and Emerging Swine Respiratory Pathogens

**DOI:** 10.3390/microorganisms14051159

**Published:** 2026-05-20

**Authors:** Nelly O. Elshafie, Rebecca P. Wilkes

**Affiliations:** 1 Willie M. Reed Animal Disease Diagnostic Laboratory, Department of Comparative Pathobiology, College of Veterinary Medicine, Purdue University, West Lafayette, IN 47907, USA; 2 Veterinary Diagnostic Laboratory, University of Kentucky, Lexington, KY 40511, USA

**Keywords:** swine respiratory disease, targeted NGS, Ion Torrent, multiplex diagnostics, pathogen surveillance

## Abstract

Respiratory disease remains one of the mostly costly challenges in the U.S. swine industry and is frequently associated with polymicrobial infections. Routine qPCR assays are highly sensitive but are limited in multiplexing capacity and generally do not provide sequencing information for pathogen characterization. We hypothesized that a target next-generation sequencing (tNGS) panel could provide the broad, simultaneous detection of swine respiratory pathogens while preserving clinically relevant sensitivity. A multiplex Ion Torrent tNGS panel was developed and analytically validated using 20 serially diluted qPCR-positive clinical samples and synthetic gBlock controls, followed by diagnostic validation with 25 qPCR positive and 25 qPCR negative respiratory samples. Most targets were detected across clinically relevant pathogens concentrations. Actinobacillus suis primers showed nonspecific amplification, streptococcus suis serotyping was not consistently achievable in clinical samples, and porcine reproductive and respiratory syndrome virus typing was limited to distinguishing North American and European genotypes. Diagnostic agreement with routine qPCR was high (Cohen’s κ = 0.84), although sensitivity decreased for low-abundance targets. The assay detected mixed infections and additional organisms outside routine qPCR panels. These findings support tNGS as a complementary diagnostic and surveillance tool for swine respiratory disease.

## 1. Introduction

Infectious disease syndromes in swine are frequently multifactorial, with clinical signs that overlap among viral, bacterial, and occasionally fungal pathogens. Porcine respiratory disease complex (PRDC) [1] exemplifies this challenge, as it commonly involves coinfections with multiple agents, making definitive diagnosis difficult using single-pathogen assays. Consequently, there is a critical need within the swine industry for comprehensive diagnostic approaches capable of simultaneously detecting co-infections and identifying emerging or unexpected pathogens.

Most veterinary diagnostic laboratories rely on real-time PCR (qPCR), bacterial and fungal culture, histopathology, immunohistochemistry, in situ hybridization, or combinations of these methods to investigate infectious disease cases. While these tools can be highly sensitive and specific, their multiplexing capacity remains limited relative to targeted next-generation sequencing (tNGS) approaches [2]. Cost considerations and sample volume constraints often restrict testing to a subset of the most common agents associated with a given clinical syndrome, thereby limiting surveillance capacity and reducing the likelihood of detecting rare or emerging pathogens. Determining which assays to apply for any individual case can be challenging, particularly when clinical presentation does not clearly indicate a specific etiologic agent.

tNGS offers a promising solution to these limitations [3,4,5]. Next-generation sequencing is a massively parallel sequencing technology that enables the simultaneous sequencing of large numbers of nucleic acid fragments, including host and pathogen genetic material [6]. tNGS enriches predefined genomic regions using PCR-based primers or capture probes, substantially increasing analytical sensitivity for pathogens of interest while reducing sequencing depth requirements and associated costs [7]. This targeted enrichment enables the detection of pathogens across a broad range of targets, with sensitivity comparable to qPCR for clinically relevant pathogen loads, while expanding the scope of detectable agents. The large number of primers that can be used allows the incorporation of strain- or variant-level information into routine diagnostic testing through the use of primers targeting these defining regions of the pathogens.

The multiplexing capacity of tNGS makes it particularly well-suited for syndromic testing, such as respiratory disease diagnostics, where numerous pathogens may contribute to disease. While tNGS platforms can theoretically include hundreds of bacterial and viral targets in a single assay, far exceeding the multiplexing capabilities of PCR panels, which are typically limited to five to eight targets per reaction, the panel developed in this study was designed to detect approximately 25 clinically relevant swine respiratory pathogens. In addition to improving diagnostic efficiency, tNGS enables surveillance for emerging or rare pathogens alongside routine diagnostic testing. Importantly, this approach allows the testing of sample types that represent herd-level infectious status, including oral fluids and pooled processing fluids, thereby enhancing population-level monitoring.

tNGS has already been successfully implemented in veterinary diagnostic laboratories for the detection of bovine [8,9] and vector-borne pathogens [10], with multiple peer-reviewed studies demonstrating its analytical and diagnostic utility [9,11]. Building on this foundation, the long-term goal of this study is to develop a comprehensive diagnostic platform for infectious agents of swine across multiple syndromes, including respiratory, enteric, neurologic, reproductive, and pathogens of One Health concern. The objective of the present study was to validate a targeted NGS panel for the identification of swine respiratory pathogens.

The core hypothesis of this study is that a multiplex tNGS assay will demonstrate diagnostic performance comparable to established qPCR assays while providing substantial added value through comprehensive pathogen detection and genomic characterization within a single test. To test this hypothesis, we pursued four specific aims: (1) development and optimization of primers targeting respiratory pathogens of swine, (2) validation of primer specificity and sequencing performance (analytical specificity), (3) determination of analytical sensitivity in comparison with qPCR, and (4) validation of diagnostic performance using clinical samples, including the assessment of diagnostic sensitivity, specificity, and agreement with existing laboratory methods (diagnostic sensitivity/specificity).

## 2. Materials and Methods

### 2.1. Assay Design and Primer Panel Development

An array of primers targeting multiple regions across the genomes of common and emerging swine pathogens was designed in collaboration with the AgriSeq Bioinformatics team (Thermo Fisher Scientific, Waltham, MA, USA). The panel was developed to enable the simultaneous detection of viral, bacterial, and selected virulence-associated targets relevant to swine disease syndromes. Multiple primer sets were included for each pathogen to improve the robustness of detection and mitigate the impact of sequence variability.

In total, the assay consists of 2176 primers (1088 amplicons), which were divided into two primer pools to maximize amplification efficiency across targets and to minimize primer–primer interactions within the highly multiplexed reactions. While this work focused on the primers for respiratory pathogens (*n* = 25) in the assay, the design also contains primers for pathogens associated with other syndromes, such as enteric and reproductive diseases. Primer design focused on highly conserved genomic regions to ensure broad detection, while selected targets included regions enabling genotyping or lineage assignment for specific viral pathogens, such as porcine reproductive and respiratory syndrome virus (PRRSV) porcine circovirus type 2 (PCV2), and porcine circovirus type 3 (PCV3), and for typing some bacterial pathogens, including *Streptococcus suis* (*S. suis*) and *Actinobacillus pleuropneumoniae* (*APP*). Target genomic regions were compiled in FASTA format, and corresponding primer target coordinates were defined in BED format and incorporated into the Torrent Suite Software (TSS; Thermo Fisher Scientific, Waltham, MA, USA) to support downstream reference-based read mapping, coverage assessment, and taxonomic assignment. These reference and annotation files were used throughout data processing and interpretation. Primer pools were synthesized by Thermo Fisher Scientific using the AgriSeq platform. Primer pools are available from Thermo FisherScientific by emailing AgriSeqOrders@thermofisher.com—design number PRD_MLS_Design_20250310. The FASTA reference sequences and BED annotation files used for primer targeting and read mapping are provided as Supplementary Data Files S1 and S2, respectively.

### 2.2. Sample Selection and PCR Testing

Stored nucleic acids (−80 °C) from clinical samples (including respiratory swabs, oral fluids, tissue pools, feces/intestinal samples), obtained from swine submitted to the Purdue University Animal Disease Diagnostic Laboratory (ADDL), an American Association of Veterinary Laboratory Diagnosticians (AAVLD)-accredited laboratory, for routine diagnostic investigation, were evaluated with the tNGS panel for respiratory syndrome testing. Samples were selected retrospectively based on results from the laboratory’s validated qPCR assays, including PRRSV, SIV, PCV2/3, *M. hyopneumoniae* (MHP), *S. suis*, *Glaesserella parasuis* (GPS), APP, and *M. hyorhinis* (MHR). A cycle threshold (Ct) value of ≤37 was interpreted as a positive result for diagnostic purposes based on the standard diagnostic cutoff used in the ADDL established through the internal validation of assay performance and avoidance of false-positive results. Clinical isolates of *Streptococcus suis*, previously serotyped in the ADDL clinical bacteriology laboratory, were also evaluated by the assay.

A total of 70 clinical samples were included: 20 positive samples to establish relative limits of detection, and an independent set of 25 qPCR-positive respiratory cases and 25 qPCR-negative case controls for determining diagnostic validation.

**Figure 1:**
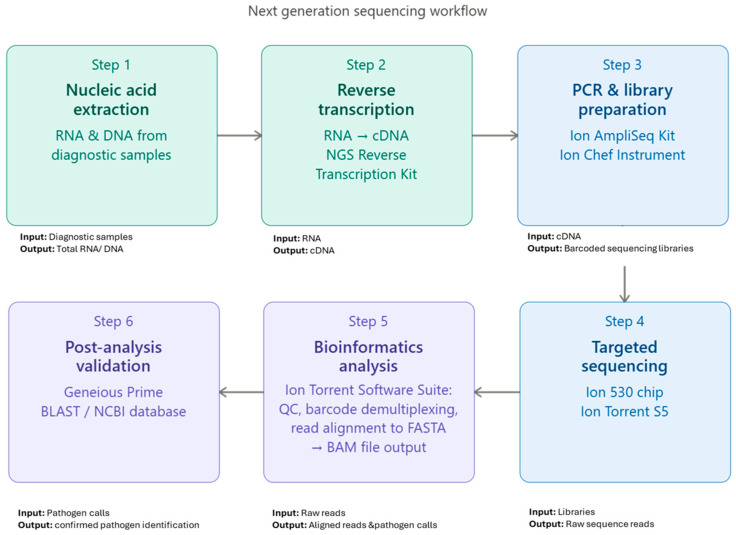
Schematic overview of the targeted next-generation sequencing workflow.

Samples represented a range of qPCR Ct values (11.43–36.94), reflecting varying pathogen loads typically encountered in diagnostic submissions. Sample selection was performed to ensure the representation of both viral and bacterial respiratory pathogens, as well as mixed infections commonly associated with porcine respiratory disease complex (PRDC). Total nucleic acids had been previously extracted using the MagMAX™ CORE Nucleic Acid Purification Kit- Protocol A (Thermo Fisher Scientific, Waltham, MA, USA) on a KingFisher™ Flex System (Thermo Fisher Scientific, Waltham, MA, USA), following the manufacturer’s instructions. Both RNA and DNA targets were included in the tNGS assay, so reverse transcription was performed for all samples using the NGS RT kit according to the manufacturer’s recommendations (Thermo Fisher Scientific, Waltham, MA, USA) (Figure 1) [12]:

(1) Nucleic acid extraction: RNA and DNA were extracted from swine diagnostic samples using validated ADDL protocol.
(2) Reverse transcription: RNA templates were reverse transcribed using the NGS RT system according to the manufacturer’s recommendations.
(3) Automated multiplex PCR and library preparation: Multiplex PCR setup and library construction were automated using the Ion AmpliSeq Library Kit for Chef DL8 on the Ion Chef Instrument.
(4) Targeted sequencing: Barcoded libraries were templated, enriched, and loaded onto the Ion 530 chip for sequencing on the Ion Torrent S5 platform.
(5) Bioinformatics analysis: Raw reads were processed using the Ion Torrent Suite Software for quality filtering and alignment.
(6) Post-analysis validation: Sequences were reviewed using Geneious Prime, and pathogen identities were confirmed by BLAST alignment against the NCBI nucleotide databas: https://blast.ncbi.nlm.nih.gov/Blast.cgi (accessed on 10 March 2025).

### 2.3. tNGS Library Preparation and Sequencing

Targeted amplification and sequencing library preparation were performed semi-automatically on the Ion Chef™ Instrument (Thermo Fisher Scientific, Waltham, MA, USA) using the Ion AmpliSeq™ Kit for Chef DL8 (Thermo Fisher Scientific, Waltham, MA, USA), according to the manufacturer’s recommendations. Two primer pools were used for the multiplex PCR, with each primer pool added at a 2× concentration. Up to eight samples per library preparation were processed simultaneously, generating individually barcoded libraries that were pooled at equimolar concentrations to produce a final library pool of approximately 100 pM.

Sequencing was performed on an Ion GeneStudio™ S5 System (Thermo Fisher Scientific, Waltham, MA, USA) using Ion 530™ chips (Thermo Fisher Scientific, Waltham, MA, USA). Chip loading was conducted on the Ion Chef using the Ion 510™, Ion 520™, and Ion 530™ Kit—Chef (Thermo Fisher Scientific, Waltham, MA, USA), with equal volumes of each pooled library combined prior to loading. Sequencing generated approximately 500,000 reads per sample, reflecting overall sequencing depth but not uniform coverage across all amplicons. Coverage was evaluated at the pathogen-target level rather than by global amplicon uniformity.

Primary data processing, including quality control, barcode demultiplexing, and reference-based read mapping, was performed using Torrent Suite Software (TSS) v5.16.1 on the Ion Torrent Server. Resulting BAM files were further evaluated using Geneious Prime v2023.2.1 for coverage assessment and read inspection. The confirmation of taxonomic assignments was performed using NCBI BLAST analysis. A detection was considered supportive of target identification when a minimum of 10 unique reads aligned with ≥90% nucleotide identity across an amplicon length of at least 100 base pairs. Detection thresholds were established empirically after reviewing sequencing results from the current dataset performance and informed by prior tNGS validations studies conducted in our laboratory. The cutoff of 10 reads was set conservatively to prevent false-positive detections, and a length of 100 base pairs was set based on off-target amplification producing nonspecific small reads.

### 2.4. Feasibility, Analytical Sensitivity and Specificity of the Assay

The feasibility of the targeted sequencing assay was evaluated using a combination of qPCR-positive clinical specimens and synthetic DNA controls (gBlocks^®^ Gene Fragments, Integrated DNA Technologies, Coralville, IA, USA) representing targeted genomic regions.

Analytical sensitivity was first assessed using 20 qPCR-positive clinical specimens representing complex, multi-pathogen respiratory infections. Selected samples contained combinations of PRRSV, PCV2, PCV3, *MHP*, *MHR*, *S. suis*, and *GPS*, as determined by the qPCR testing. These samples were subjected to sequential serial dilutions and re-tested by the tNGS assay to evaluate the consistency of detection as pathogen concentration decreased, representing a range of qPCR cycle threshold (Ct) values (11.43–36.94). Analytical sensitivity was further evaluated using synthetic gBlock DNA controls representing selected respiratory pathogens, including targets that are rare, emerging, or not routinely available as positive clinical material in the United States. Two gBlocks per pathogen were used, which reflected regions targeted by different primer sets in the assay. Ten-fold serial dilutions of gBlocks were used to assess primer performance and sequencing detection independent of clinical matrix effects. The gblock DNA-based controls did not account for RT efficacy for RNA viruses; thus, their use offers a proof of concept for rare target detection rather than full analytical validations for RNA pathogens.

Primer specificity was addressed at multiple stages. During assay development, in silico evaluations were performed to minimize cross-reactivity and off-target amplification. Following sequencing, amplified products generated by each primer set were further assessed through sequence similarity searches against the NCBI nucleotide database using BLAST algorithms.

### 2.5. Diagnostic Sensitivity and Specificity (Positive and Negative Percent Agreement)

tNGS results were interpreted in comparison with findings from the routine qPCR assays used as the reference method. Positive samples (N = 25, Ct values ranging from 7.82–37) were defined as those from pigs with respiratory clinical signs testing positive for one or more respiratory pathogens. These samples were distinct from the samples used for analytical testing. Negative control samples (N = 25) consisted of submissions that tested negative for all targets in a respiratory PCR panel (PRRSV, SIV, PCV2/3, and MHP). These negative samples were swine fecal samples and were chosen as negative controls instead of negative respiratory samples, to avoid the detection of respiratory pathogens included in our design in “negative controls”. Agreement between tNGS and PCR was assessed at the sample level using standard diagnostic concordance metrics, including positive percent agreement (PPA), negative percent agreement (NPA), and overall accuracy. Confidence intervals were calculated using the exact (Clopper–Pearson) method. In addition, Cohen’s kappa (κ) statistics were calculated to quantify the level of categorical agreement beyond chance. These measures were used to evaluate the diagnostic performance of the tNGS assay relative to established qPCR testing across positive and negative clinical samples.

## 3. Results

### 3.1. Panel Design and Primer Development

A comprehensive multiplex primer pool targeting swine pathogens, including approximately 25 respiratory pathogens, was successfully developed in collaboration with Thermo Fisher Scientific’s AgriSeq bioinformatics team. The finalized panel encompassed major viral and bacterial respiratory pathogens, including PRRSV, SIV, PCV2, PCV3, *MHP*, *MHR*, *Pasteurella multocida*, and *GPS*, as well as emerging or less routinely tested agents such as adenoviruses, bocaviruses, and porcine parainfluenza 1, or non-endemic or exotic pathogens like Nipah and Sendai incorporated for surveillance preparedness, with validation limited to synthetic gBlock controls. In silico and empirical optimization of primer pools resulted in uniform annealing performance across a pathogen, minimal primer–primer interactions, and no evidence of any systematic off-target amplification of porcine host nucleic acids.

### 3.2. Analytical Specificity

Successful amplification and sequencing were achieved for the respiratory pathogens included in the assay, except *Actinobacillus suis*, for which we lacked a positive clinical sample to confirm the ability of the panel to detect this pathogen (Table 1). Pathogen detection was confirmed using either corresponding clinical samples or synthetic DNA controls (gBlocks) when clinical material was unavailable. Sequence reads shorter than 100 bp were excluded from analysis and classified as false priming events.

Based on primer design, expected amplicon sizes were approximately 200 bp. The BLAST analysis of trimmed and aligned sequence reads generated by Torrent Suite Software (TSS) confirmed primer specificity for the intended targets in most cases. However, sequences detected with *Actinobacillus suis* (*A. Suis*) primers produced reads that BLAST analysis demonstrated were an amplification of nonspecific genomic fragments, most commonly corresponding to *Glaesserella* or *Streptococcus species*, indicating insufficient specificity for this target under the current assay configuration. For *S. suis*, genetic serotyping was possible for all serotypes when testing with clinical isolates, but this was not possible with *S. suis* detected in clinical samples. However, the assay could differentiate between different APP capsules and apx sequences for typing in clinical samples containing APP serotype 2.

The tNGS assay successfully differentiated North American (Type 2) and European (Type 1) PRRSV strains based on a sequence analysis of conserved genomic regions included in the primer panel. This distinction was consistently observed across PRRSV-positive clinical samples and was concordant with available qPCR results. Although multiple linages were incorporated into the panel design, PRRSV type 2 sequence could not be reliably assigned to a specific lineage, as the targeted regions amplified conserved sequence shared across multiple lineage groups, with the drop-out of some specific regions, limiting lineage-level discrimination.

For PCV, the targeted NGS assay enabled genotype-level discrimination, including the reliable identification of PCV2 genotype D and differentiation from other PCV2 genotypes represented in the assay. For clinical samples for which SIV could be detected, subtyping was possible.

### 3.3. Analytical Sensitivity

#### 3.3.1. Analytical Sensitivity Using Serially Diluted Clinical Samples

Across multiple dilutions, the tNGS assay consistently detected major respiratory pathogens, including PRRSV type 1 and type 2, PCV2, PCV3, and MHP, demonstrating robust performance in complex clinical matrices. As expected, read depth declined with increasing dilution; however, the qualitative detection of key pathogens was maintained even at low read counts. For example, PCV2 remained detectable at approximately 30 reads ≈ Ct 33.65 in the highest dilution tested. These findings indicate that a loss of individual targets at higher dilutions reflects biological variability and stochastic sampling effects inherent to low-input sequencing rather than systematic assay failure.

#### 3.3.2. Approximate Qualitative Detection Thresholds Based on Ct Values

To contextualize analytical sensitivity relative to routine diagnostics, PCR cycle threshold (Ct) values associated with the highest dilutions at which pathogens remained detectable by tNGS were summarized (Table 2). The number of independent samples contributing to each estimate is provided in Table 2. These values represent approximate analytical limits of detection. Only one subtype of SIV was used for analytical sensitivity testing and was detected at a Ct value of approximately 29.09; this should be interpreted as representative, rather than a broader generalization that this would be the approximate LOD for all subtypes of SIV. The approximate LOD observed for *APP* was near the qPCR detection limit, but the tNGS detection at this level was considered a true-positive detection based on the sequences meeting the required parameters for a positive detection.

For most pathogens, approximate detection thresholds were derived from a single representative clinical sample; however, multiple independent samples were available for PRRSV, PCV2, PCV3 and SIV.

Across evaluated pathogens, the limit of detection by tNGS was observed at Ct values ranging from approximately 28 to 35, depending on the organism. Viral targets such as PRRSV, PCV2, and PCV3 remained detectable at Ct values approaching or exceeding 30, while bacterial pathogens, including *Pasteurella multocida* and *GPS*, were detectable at Ct values up to approximately 33. The detection of *S. suis* was observed at Ct values near 28, consistent with lower abundance recovery in complex clinical matrices.

#### 3.3.3. Analytical Sensitivity Using Synthetic gBlock Controls

Viral targets (represented by the gBlocks) were consistently detected between 100 and 1000 copies (Table 3), representing a proof of concept for target detection rather than full analytic sensitivity for RNA viruses, as RT efficacy was not evaluated. Differences in read counts between gene targets for the same pathogen reflect expected variation in primer efficiency and amplicon performance rather than a loss of detection. The reported detection limits correspond to the lowest tested concentrations; additional dilution steps were not performed to determine the absolute lower bound of detection.

Together, these clinical dilution experiments complement the gBlock-based analytical sensitivity studies and confirm that the tNGS respiratory panel exhibits strong analytical sensitivity in both synthetic controls and complex clinical matrices. While the tNGS assay is qualitative rather than quantitative, detection limits were generally comparable to qPCR for clinically relevant targets, with reduced sensitivity observed primarily at Ct values above approximately 30 for most of the pathogens.

#### 3.3.4. Diagnostic Sensitivity and Specificity (Positive and Negative Percent Agreement)

Across the 50 samples that had been previously characterized using the laboratory’s routine porcine qPCR panels (25 PCR-positive respiratory cases and 25 negative controls) (Supplementary Tables S1 and S2), tNGS demonstrated high overall concordance with qPCR. Among qPCR-positive samples, 21/25 showed complete agreement between PCR and tNGS for all targeted respiratory pathogens, including viral agents (PRRSV, PCV2, PCV3, and SIV) and bacterial agents (MHP), while 4/25 (Samples 1, 18, 21, 25—Supplementary Table S1) did not. Notably, all discordant results occurred at high Ct values (30.97–36.94), consistent with low target abundance near the limit of detection. tNGS identified multiple pathogens that were not detected by routine PCR testing because they were outside the scope of the standard Porcine Respiratory Panel offered at the ADDL. These additional detections included porcine cytomegalovirus, porcine astrovirus, porcine adenovirus B, bocavirus 3, and kobuvirus, in samples already positive for one or more primary respiratory pathogens. The negative control cohort remained negative for respiratory pathogens by tNGS. Organisms considered part of the normal respiratory microbiota were excluded from evaluation. Agreement between qPCR and the Ion Torrent-targeted NGS assay was assessed using a subset of 50 samples, including 25 qPCR-positive and 25 qPCR-negative cases. Four qPCR-positive samples were not detected by Ion Torrent, while no false-positive results were observed among PCR-negative samples. The overall agreement observed was 92%, with a Cohen’s kappa coefficient of 0.84, indicating almost perfect agreement between methods for the targets included in the routine qPCR panel only (Table 4).

## 4. Discussion

This study demonstrates that a multiplex tNGS assay can function as a reliable and informative diagnostic tool for swine respiratory disease, with performance closely aligned to established qPCR testing while offering substantially broader pathogen coverage and genomic insight, and simultaneously overcoming many of their inherent limitations.

Across the clinical validation cohort, the tNGS panel showed strong concordance [13] with routine qPCR testing, reflected by an overall agreement of 92% and a Cohen’s kappa value of 0.84, indicating near-perfect categorical agreement beyond chance. However, this study represents a small number of retrospective clinical samples received at a single diagnostic laboratory, which may reduce the generalizability of these results.

Importantly, all samples that were qPCR-negative for the routine respiratory panel also tested negative by tNGS for those same targets, underscoring the assay’s high specificity and low risk of false-positive reporting. This is a critical consideration for diagnostic laboratories, where false positives can lead to unnecessary interventions, economic loss, and the erosion of producer confidence. The detection of organisms outside of the scope of this work in the negative sample set relates to the incorporation of primers in the panel for enteric pathogens and the use of fecal samples for negative controls.

A limitation of the study was the lack of a positive sample for *A. suis* and thus adequate evaluation of the specificity for the detection of that organism. The primers for *A. suis* were discovered to detect bacteria that were not *A. suis*; however, with an evaluation of the sequences obtained from these primers, *A. suis* should be able to be differentiated from these other bacteria, but that could not be confirmed.

Discrepancies between methods were limited to qPCR-positive samples and were consistently associated with targets detected at high Ct values, indicating low pathogen abundance. These findings are consistent with expected stochastic effects when sequencing low-copy templates in highly multiplexed assays. Rather than reflecting systematic assay failure, these occasional missed detections likely result from random sampling limitations near the analytical detection threshold. Some targets—specifically, SIV and respiratory coronavirus—performed below expectations, based on relative detection limits. For the RNA viruses, this is likely due to lack of adequate primers to detect sequence differences. Future optimization should include additional primers to provide better coverage for variant sequences. While the design of primers for qPCR assays for the detection of conserved regions of RNA viruses is relatively easy, this is more difficult for tNGS, which has more rigid criteria for design, such as generating PCR products that are generally 250 bp to be sequenced with this platform.

The evaluation of analytical sensitivity using serially diluted clinical samples confirmed that the tNGS assay retains robust detection capability across a wide range of pathogen concentrations, including those commonly encountered in routine diagnostic submissions. Viral agents central to porcine respiratory disease complex, such as PRRSV, PCV2, and PCV3, remained detectable at Ct values near or slightly above 30. Several bacterial pathogens, including *Pasteurella multocida* and *GPS*, were also recovered at relatively high Ct values, demonstrating the effective amplification and sequencing of low-abundance bacterial targets in mixed infections. The assay consistently detected *S. suis* in multiple clinical specimens; however, serotype-level discrimination in clinical samples was not reliably achieved. This limitation is likely attributable to low bacterial abundance and the secondary nature of *S. suis* infections in samples dominated by primary viral pathogens. In such cases, sequencing depth and competition among targets within the highly multiplexed assay likely limited the recovery of sufficient discriminatory sequences for serotyping. Additionally, retrospective samples were used, which were extracted without bead beating. Future studies would incorporate bead-beating steps during extraction to improve nucleic acid recovery and potentially enhance bacterial detection. This may have reduced the amount of the Gram-positive *S. suis* detected in the clinical samples. Bead beating during the extraction process may help to enhance the detection of this bacterium to allow for increased discrimination. Accordingly, *S. suis* detections were conservatively reported at the species level.

The consistent detection of multiple agents within the same sample highlights one of the most significant strengths of the tNGS approach: its capacity to resolve complex coinfections without requiring prior assumptions about the causative agent. In syndromes such as PRDC, where disease severity and treatment outcomes are often influenced by pathogen interactions rather than single agents, this capability represents a substantial diagnostic advantage.

The detection of additional organisms in the positive samples used for diagnostic sensitivity testing does not represent discordance between methods but rather reflects the broader analytical coverage of the targeted NGS assay. These findings demonstrate the added value of tNGS for comprehensive pathogen detection, co-infection characterization, and the surveillance of emerging or less routinely tested agents that are not captured by conventional multiplex qPCR panels. However, the detections in these samples were not confirmed by other methods. Additionally, these results should not be overinterpreted, as some of these organisms, such as porcine cytomegalovirus and adenovirus B, are ubiquitous in the swine population, and primers for these organisms were included for testing other disease syndromes. However, these organisms are considered part of the porcine respiratory disease complex. Porcine astrovirus 4 was also detected in this sample set. The primers were incorporated for enteritis, but this organism is also associated with respiratory disease. The detection of kubovirus in a respiratory sample was unexpected. The detection of astrovirus 4 and bocavirus 3, also in this sample (sample 14, Supplementary Materials) suggests there may have been some fecal contamination of this respiratory sample.

Analytical sensitivity testing using synthetic gBlock controls further validated primer performance and sequencing reliability independent of biological sample variability. These experiments were particularly valuable for targets that are rare, emerging (e.g., Sendai virus), or not encountered in U.S. swine populations (e.g., Nipah virus). The detection of most viral targets at low copy numbers confirms that the assay is well-positioned for the early recognition of novel or re-emerging respiratory pathogens before they become widespread. However as previously mentioned, for RNA viruses, gBlocks do not fully capture the complexity of RNA templates or the reverse transcription step and thus should be considered as an initial analytical assessment rather than complete validation.

From a surveillance perspective, this capacity is highly relevant. The ability to incorporate uncommon or emerging agents into a single assay that could be used routinely enables proactive monitoring rather than reactive testing, strengthening biosecurity and preparedness at both the herd and regional levels [12,14,15].

Beyond simple presence or absence, the targeted NGS (tNGS) panel was designed to include genotype and strain, informative genomic regions for selected viral pathogens of interest. This design enabled additional genetic resolution for several viruses, including the differentiation of PRRSV North American (Type 2) versus European (Type 1) genotypes, the identification of PCV2 genotype D versus non-D genotypes, and the determination of SIV subtypes when SIV was detected. Nevertheless, it is important to note that SIV subtyping was not fully validated with all the subtypes or with samples from multiple locations.

However, full lineage-level discrimination was not consistently achieved for all targets, particularly for PRRSV in clinical specimens. Although lineage-associated regions were incorporated into the primer design, sequence coverage across lineage-defining regions was frequently insufficient in mixed infections or low-viral-load samples to support confident lineage assignment. Additionally, we did not have samples representing all PRRSV Type II lineages, so a full evaluation could not be performed. As a result, PRRSV results were conservatively reported at the genotype level rather than at the lineage level. These findings indicate that, while the current assay reliably detects PRRSV presence, an additional optimization or redesign of lineage-targeting primers may be required to consistently achieve lineage-level resolution, which was an intended objective of the panel design.

The sequence-level data generated by the tNGS approach supports an improved interpretation of circulating viral diversity and may aid outbreak investigations and epidemiologic assessments when sufficient sequence coverage is achieved. Given the large number of primers that can be incorporated into these types of assays, the addition of primers is possible as new pathogens are discovered or pathogen sequences change. Equally important is the assay’s compatibility with herd-level sample types, including pooled and population-representative specimens (oral fluids). This expands its utility from individual case diagnosis to broader monitoring programs, where understanding pathogen diversity and circulation patterns is often more informative than identifying a single agent in a single animal [14].

For pork producers, the immediate benefit of this tNGS approach lies in its ability to provide a more complete picture of respiratory disease dynamics from a single test [14]. By identifying multiple pathogens simultaneously and revealing underlying coinfections, the assay can support more informed treatment decisions, guide targeted interventions, and reduce reliance on sequential or repeated testing. This is particularly valuable in situations where clinical signs are nonspecific or when initial diagnostics fail to fully explain disease severity or persistence.

Collectively, these findings support the role of targeted NGS as a powerful complement to, rather than a replacement for, qPCR. qPCR remains indispensable for the rapid, high-throughput detection of specific agents, particularly in acute diagnostic scenarios. However, tNGS adds a critical layer of breadth and resolution that is difficult to achieve with conventional detection assays alone. When integrated thoughtfully into diagnostic workflows, tNGS can extend laboratory capability beyond hypothesis-driven testing toward comprehensive, syndromic investigation.

## 5. Conclusions

This study demonstrates that a multiplex tNGS assay can serve as a reliable and informative diagnostic tool for detecting respiratory pathogens in swine, particularly when evaluated within the scope of targets included in routine qPCR panels. High agreement between tNGS and qPCR was observed for shared targets, supporting the analytical performance of the assay. tNGS enabled the detection of additional pathogens not included in the routine qPCR panels, highlighting its potential to expand diagnostic coverage. However, the resolution of strain- and lineage-level information was not consistently achievable across all targets in clinical samples and was dependent on sequence coverage and target abundance. Limitations of this study include the use of retrospective samples processed without bead beating, the incomplete exploration of detection limits, no intra- or inter-assay repeatability studies, and the use of synthetic gBlock controls that do not fully replicate RNA virus detection. Future studies should incorporate optimized extraction methods, along with intra-assay and inter-assay repeatability analyses, to further characterize assay precision reproducibility, in addition to an expansion of the validation using larger and multi-center cohorts and refine primer design for underperforming targets. Overall, multiplex tNGS represents a promising complementary approach to routine diagnostics, with the potential to enhance pathogen detection and surveillance in swine respiratory disease.

## 6. Simple Summary

Respiratory disease is a major and costly problem for U.S. pig producers. It is especially challenging because sick pigs are often infected with more than one virus or bacterium at the same time. Most current diagnostic tests look for only one pathogen or a small group of pathogens at a time and do not provide detailed genetic information that helps to track how diseases spread or change.

This project developed a new laboratory test that can detect many important swine respiratory pathogens at once using targeted next-generation sequencing. The test was evaluated using both clinical samples and laboratory controls and showed strong agreement with commonly used PCR tests. Importantly, it was able to identify mixed infections and detect organisms that may be missed by routine testing.

While the new test is not intended to prove that pigs are completely disease-free and may still need refinement for some pathogens, it shows clear value as a complementary diagnostic and surveillance tool. Using this approach can improve outbreak investigations, guide vaccine decisions, and strengthen the monitoring of swine respiratory health.

## Figures and Tables

**Table 1:** Etiological agents evaluated for analytical specificity.

Agent *	Source	Detected	Specificity/Notes
Nipah virus	gBlock	Yes	Analytical detection only
Porcine hemagglutinating encephalomyelitis virus (PHEV)	gBlock	Yes	Analytical detection only
Rubulavirus	gBlock	Yes	Analytical detection only
Porcine parvovirus 2	gBlock	Yes	Analytical detection only
Porcine bocavirus 1	gBlock	Yes	Analytical detection only
Orthoreovirus	gBlock	Yes	Analytical detection only
Porcine parainfluenza virus 1	gBlock	Yes	Analytical detection only
Porcine circovirus 4 (PCV4)	gBlock	Yes	Analytical detection only
Sendai virus	gBlock	Yes	Analytical detection only
Influenza C virus	gBlock	Yes	Analytical detection only
Influenza D virus	gBlock	Yes	Analytical detection only
Porcine adenovirus (PAdV)	gBlock	Yes	Analytical detection only
PRRSV Type 1	Clinical sample (Ct = 17.38)	Yes	Genotype-level resolution achieved
PRRSV Type 2	Clinical sample (Ct = 12.5)	Yes	Genotype-level only; lineage not consistent
Porcine circovirus 2 (PCV2d)	Clinical sample (Ct = 14.74)	Yes	Genotype-level discrimination achieved
Porcine circovirus 3 (PCV3)	Clinical sample (Ct = 15.3)	Yes	Reliable detection
Porcine respiratory coronavirus (PRCV)	Clinical sample (Ct = 25.57)	Yes	Reliable detection
Swine influenza virus (H1N2)	Clinical sample (Ct = 18.21)	Yes	Subtyping possible in high-quality sample; did not evaluate all subtypes of SIV
*Mycoplasma hyopneumoniae* (*MHP*)	Clinical sample (Ct = 27.55)	Yes	Reliable detection
*Mycoplasma hyorhinis* (*MHR*)	Clinical sample (Ct = 25.51)	Yes	Reliable detection
*Actinobacillus pleuropneumoniae* (*serotype 2*)	Clinical sample (Ct = 28.68)	Yes	Reliable detection
*Glaesserella parasuis*	Clinical sample (Ct = 27.78)	Yes	Reliable detection; primers for *Actinobacillus suis* also detected this pathogen
*Bordetella bronchise ptica*	Clinical sample (Ct = 29.26)	Yes	Reliable detection
*Streptococcus suis*	Clinical sample (Ct = 25.52)	Yes	Species-level only; serotyping not reliable in clinical sample; *Actinobacillus suis* primers also detected this pathogen
*Pasteurella multocida*	Clinical sample (Ct = 28.07)	Yes	Reliable detection

* All the etiological agents were detected by tNGS assay except *Actinobacillus suis* (*A. suis*), which showed cross-reactivity and could not be reliably confirmed. Primer sets for all respiratory pathogens included in the assay were tested with known positive samples or sequences representing the expected pathogen, except those for *A. suis* (no positive *A. suis* sample tested).

**Table 2:** Approximate qualitative detection thresholds (compared to Ct values from qPCR testing) for major PRDC-associated pathogens based on serial dilution of PCR-positive clinical samples (*n* = 20).

Agent	Approximate Ct at Detection Limit	*n*
Porcine Reproductive and Respiratory Syndrome Virus (PRRSV)	30.12	4
Porcine Circovirus Type 2 (PCV2)	33.65	3
Porcine Circovirus Type 3 (PCV3)	30.59	3
Swine Influenza Virus A (SIV) (Sanger sequencing confirmed H1N2, which was also identified by tNGS assay)	29.09	2
Porcine Respiratory Coronavirus (PRCV)	28.87	1
*Mycoplasma hyopneumoniae* (*MHP*)	31.54	1
*Mycoplasma hyorhinis* (*MHR*)	30.06	1
*Actinobacillus pleuropneumoniae* (*APP*)	35.16	1
*Glaesserella parasuis*	32.92	1
*Bordetella bronchiseptica*	32.57	1
*Streptococcus suis*	28.6	1
*Pasteurella multocida*	32.98	1

*n* indicates the number of independent clinical samples contributing to each approximate qualitative detection threshold. For pathogen represented by a single sample, the reported value should be interpreted as an estimate rather than a clear cutoff analytic limit of detection.

**Table 3:** Copy number-based detection limits of synthetic gBlocks representing targets, reported as copies per reaction based on serial dilutions.

Pathogen-ID	Detection Limit (Based on Results from Best-Performing Primers in the Assay) *
Nipah	100 copies
Porcine hemagglutinating encephalomyelitis virus (PHEV)	1000 copies
Rubulavirus	100 copies
Parvovirus 2	100 copies
Porcine bocavirus 1	100 copies
Orthoreovirus	1000 copies
Porcine Parainfluenza 1	1000 copies
Porcine Circovirus 4	100 copies
Sendai virus	100 copies
Influenza C	100 copies
Influenza D	100 copies
Porcine Adenovirus B	1000 copies

* These values reflect the lowest tested gBlock concentrations detected using the best-performing primer sets and should be interpreted as proof-of-concept analytical detection limits rather than absolute lower limits of detection.

**Table 4:** Two-by-two contingency table summarizing categorical agreement between tNGS and qPCR for the respiratory pathogens included in the routine qPCR panel, used for Cohen’s kappa analysis.

	tNGS Positive	tNGS Negative	Total
qPCR Positive	21 *	4	25
qPCR Negative	0	25 **	25
Total	21	29	50

* Positive percent agreement was 84.0% (21/25; 95% CI: 63.9–95.9%), and ** negative percent agreement was 100% (25/25; 95% CI: 86.3–100%). Confidence intervals were calculated using the exact (Clopper–Pearson) method. Cohen’s kappa coefficient was 0.84 (almost perfect agreement).

## Data Availability

The data presented in this study are available within the article and its Supplementary Materials. Sequence data can be provided on request.

## Supplementary Materials

The following supporting information can be downloaded at: https://www.mdpi.com/article/10.3390/microorganisms14051159/s1, Data File S1; FASTA reference sequences used for target identification, Data File S2; BED annotation coordinates for all assay targets. Table S1: Comparison of PCR and targeted NGS (Ion Torrent) results for PCR-positive clinical samples; Table S2: Targeted NGS (Ion Torrent) results for PCR-negative respiratory control samples.
